# Protumorigenic Role of Elevated Levels of DNA Polymerase Epsilon Predicts an Immune-Suppressive Microenvironment in Clear Cell Renal Cell Carcinoma

**DOI:** 10.3389/fgene.2021.751977

**Published:** 2021-12-07

**Authors:** Xiaohui Wu, Haijia Tang, Wen-Hao Xu, Haidan Tang, Shiyin Wei, Aihetaimujiang Anwaier, Haineng Huang, Yuan-Yuan Qu, Hailiang Zhang, Shuai Zhao, Hui Li, Wangrui Liu, Hongjing Chen, Chen Ding, Dingwei Ye

**Affiliations:** ^1^ Department of Urology, Fudan University Shanghai Cancer Center, State Key Laboratory of Genetic Engineering, Collaborative Innovation Center for Genetics and Development, School of Life Sciences, Institute of Biomedical Sciences, and Human Phenome Institute, Fudan University, Shanghai, China; ^2^ Department of Integrated Medicine, Nanjing University of Chinese Medicine, Nanjing, China; ^3^ Affiliated Hospital of Youjiang Medical University for Nationalities, Guangxi, China; ^4^ Department of Transplantation, Xinhua Hospital Affiliated to Shanghai Jiao Tong University School of Medicine, Shanghai, China; ^5^ Department of Endocrinology, Changhai Hospital, Naval Medical University, Shanghai, China; ^6^ Affiliated Maternity and Child Health Care Hospital of Nantong University, Nantong, China

**Keywords:** clear cell renal cell carcinoma, polymerase epsilon, biomarker, tumor immune microenvironment, prognosis, bioinformatics

## Abstract

Increasing evidence indicates that DNA polymerase epsilon (POLE), which mediates DNA damage repair, is significantly associated with tumor prognosis. This study aimed to analyze POLE expression in tumor samples and its prognostic value for patients with clear cell renal cell carcinoma (ccRCC). We found significantly elevated POLE expression in ccRCC tissues compared with normal tissues of multiple independent cohorts. The POLE expression levels of 523 patients with ccRCC (The Cancer Genome Atlas RNA-seq data) and 179 patients with ccRCC with immunohistochemical data (Fudan University Shanghai Cancer Center) were analyzed to investigate the prognostic implications of POLE expression. Cox regression analyses were implemented to explore the effect of POLE expression on the prognosis of pan-cancer. These findings revealed that elevated POLE expression levels significantly correlated with shorter overall survival (*p* < 0.001, *n* = 701) of patients with ccRCC. These data indicate that POLE expression may serve as a prognostic biomarker for cancers. Although POLE mutations were not significantly associated with survival benefits conferred upon patients with ccRCC, a CD4^+^ T cell-regulated immune microenvironment was significantly activated. Moreover, we found that POLE expression in cancers significantly correlated with an immunosuppressive tumor microenvironment, higher intratumoral heterogeneity, and expression of immune checkpoint genes PDCD1, CTLA4, and CD86, possibly mediated via the JAK/STAT and Notch signaling pathways. In conclusion, the present study is the first to our knowledge to indicate that elevated POLE expression is significantly associated with poor survival and an immune-suppressive tumor microenvironment in ccRCC. These findings suggest that POLE can serve as a biomarker for guiding molecular diagnosis and facilitating the development of novel individual therapeutic strategies for patients with advanced ccRCC.

## Introduction

Renal cell carcinoma (RCC) is a common urologic malignancy, accounting for 3% of all malignant tumors (Siegel et al., 2020), and its incidence is increasing annually by 3%, reaching 8–10 million cases worldwide, twice as great as 10 years ago. Clear cell RCC (ccRCC) is the most common and highly malignant pathological type of kidney cancer, accounting for 70%–85% of RCC (Capitanio and Montorsi, 2016). Approximately 25%–30% of patients with ccRCC present with metastasis upon initial diagnosis, and the 5-year survival rate of metastatic ccRCC is 32%. Increasing evidence shows that multitargeted therapies employing tyrosine-kinase inhibitors, vascular endothelial growth factor inhibitors, and immune checkpoint therapies (ICTs) confer prolonged survival benefits upon patients with advanced ccRCC (Choueiri and Motzer, 2017). However, only limited clinical treatment options are available for such patients. Previously, heterogeneous patients’ characteristics led to the rapid development of optimized treatment (Escudier et al., 2017a; Wettersten et al., 2017). Thus, it is vital to identify potential biomarkers or to develop techniques to facilitate early diagnosis and to predict prognosis with the aim of enhancing individualized treatment of patients with ccRCC.

The DNA damage repair (DDR) pathways comprise multiple interconnected cellular signaling pathways that are activated in response to DNA damage. These interlinked pathways coordinate a cascade of events, including cell cycle arrest, regulation of DNA replication, and the repair or bypass of DNA damage (Harper and Elledge, 2007). Specifically, the molecules responsible for the defects in DDR serve as targets of therapy as well as for ICTs (Pearl et al., 2015). The main DDR pathways include base excision repair, mismatch repair, nucleotide excision repair, homologous recombination repair, and nonhomologous end-joining repair. DDR regulates the STING pathway, increases the levels of chemokines including CXCL10 and CCL5, and promotes the activation of cytotoxic T lymphocytes, which in turn triggers the response of small-cell lung cancer cells to immunotherapy (Galsky et al., 2018; Sen et al., 2019). Therefore, it is important to understand the underlying functions of DDR, particularly those associated with oncogenesis and treatment (Brandsma et al., 2017).

The gene encoding the catalytic subunit of DNA polymerase epsilon (POLE), located on human chromosome 12q24.3, mediates DNA repair and chromosomal DNA replication. POLE maintains genetic stability during DNA replication and is associated with tumorigenesis, overall survival (OS) of patients, and the efficacies of antitumor immunity therapies for endometrial and colorectal cancers (Guenther et al., 2018). Furthermore, the antitumor immune response is activated in cancer patients with POLE mutations who are benefited by immuno-oncology treatment (Liu et al., 2018).

DDR coordinates the mechanisms of DNA repair and apoptosis, which regulate the responses of patients with cancer who are administered radiotherapy, chemotherapy, and targeted therapy. Thus, DDR may serve as a promising target for novel antitumor treatment strategies and for identifying gene expression signatures that predict prognosis (van Stuijvenberg et al., 2020). Recently, new ICTs, represented by PD-1/PD-L1 and CTLA4 inhibitors, have risen rapidly in the field of renal cancer treatment and achieve encouraging effects on patients with advanced treatment-refractory disease (Escudier et al., 2017b). The effects of POLE expression on the progression and prognosis of cancers provide a target for drug therapy, to achieve individualized precise treatment. However, the prognostic role and predictive relevance of POLE in ccRCC are unknown. We reasoned therefore that the important role of POLE in DDR, which may be closely related to the development of ccRCC, should be exploited as a powerful biomarker in cancer diagnosis and prognosis.

## Materials and Methods

### Patients and Tissue Samples

The large-scale cohorts included RNA sequencing data and accompanying clinicopathological data for 530 patients with ccRCC obtained from The Cancer Genome Atlas (TCGA; http://www.cancer.gov) and 178 patients with ccRCC from four independent ccRCC cohorts (Gumz Renal, Cutcliffe Renal, Lenburg Renal, and Jones Renal (Jones et al., 2014)) of the Oncomine (http://www.oncomine.com) database. The threshold was set as follows: *p*-value <0.05, gene rank >10%, Data type: mRNA, Cancer vs. Normal.

The Fudan University Shanghai Cancer Center (FUSCC, Shanghai, China) cohort comprises 179 patients diagnosed with ccRCC in its Department of Urology from April 2005 to September 2015. Electronic medical records or pathology reports provided clinicopathological data. Samples of ccRCC and normal kidney tissues collected during surgery were processed and stored at the FUSCC tissue bank.

### Immunohistochemical Analysis

Immunohistochemical (IHC) analysis was performed using an anti-POLE-N-terminal antibody (ab226848, Abcam, Cambridge, UK) diluted at 1:500. IHC was implemented in accordance with the supplier’s instructions (Xu et al., 2020a). On the basis of the integration of the degree of intensity and density of staining, two independent pathologists evaluated the overall IHC score (from 0 to 12), as follows: negative staining, 0 to 4; and positive staining, 6 to 12.

### Differential Polymerase Epsilon Expression and Survival Analysis

To evaluate the statistical significance of differential POLE expression between tumor and normal tissues, Student’s t-test was implemented. The Kaplan–Meier method (95% CI), log-rank test, and Cox regression analyses were employed to assess the significance of benefits conferred upon disease-specific survival (DSS) and OS of a separate POLE expression group and all subgroups classified according to tumor microenvironment infiltration characteristics. The latter were assessed using the Kaplan–Meier Plotter (http://kmplot.com/analysis/index). The best-performing threshold is computed and used as a cutoff value according to online webtools. Statistical analyses were performed using SPSS software (version 23.0, SPSS Inc., Chicago, IL, USA), GraphPad Prism 8.0, R software (version 3.4.3), or online webtools. All hypothetical analyses were two-sided, and *p* < 0.05 indicated a significant difference.

### Construction of a Protein–Protein Interaction Network

ProteomeHD software was used to detect coregulated proteins expressed at levels above a cutoff value. A search tool for the retrieval of interacting genes (STRING, http://string-db.org; version 10.0) was used to construct a protein–protein interaction (PPI) network of differentially expressed genes (DEGs) associated with POLE expression.

### Functional Annotations of the Polymerase Epsilon-Associated Protein–Protein Interaction Network

Functional enrichment analysis including biological processes (BPs), molecular functions (MFs), and cellular components (CCs) from the Gene Ontology (GO) Kyoto Encyclopedia of Genes and Genomes (KEGG) as well as Reactome pathway enrichment analyses were performed using large-scale biological molecular datasets. The functional-enrichment database WEB-based GEne SeT AnaLysis Toolkit (WebGestalt; http://www.webgestalt.org/; version 6.8) was employed to evaluate potential function enrichments of POLE-related gene panels (Liao et al., 2019).

### Mutation Abundance and Frequency Polymerase Epsilon Expression in Clear Cell Renal Cell Carcinoma

We analyzed mutation abundance and frequency of POLE expression in ccRCC using cBioportal for cancer genomics (http://www.cbioportal.org/) to identify significant associations between types of DNA copy number alterations of POLE and mRNA expression levels. Genes expressed at significantly elevated levels in the POLE-altered and POLE-unaltered groups were screened and identified using the Limma R package.

### Immune Infiltration Analysis of the Tumor Microenvironment

Tumor Immune Estimation Resource 2.0 (TIMER 2.0, http://timer.cistrome.org/) was used to comprehensively analyze the correlation between abundance of tumor-infiltrating immune cells and POLE expression, along with Spearman’s test. R software was used to evaluate tumor and immune system interactions among immune checkpoint molecules and tumor-infiltrating lymphocytes across human cancers from TCGA data. Spearman’s test was used to evaluate the relationship between POLE and tumor purity using the ESTIMATE algorithm (Xu et al., 2019).

## Results

### Significantly Elevated Expression of Polymerase Epsilon mRNA in Clear Cell Renal Cell Carcinoma Compared With Normal Tissues

**FIGURE 1:**
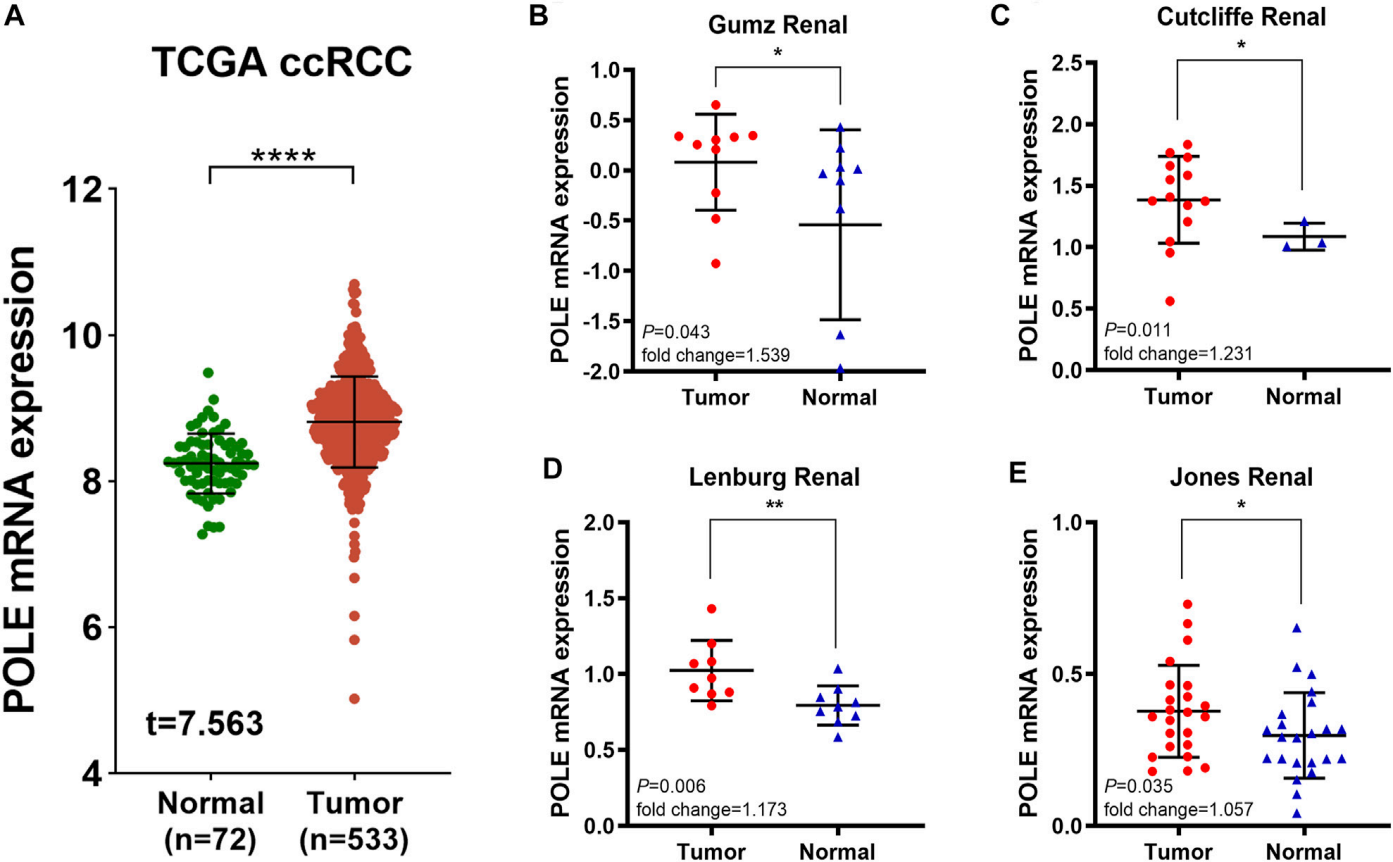
Significantly elevated polymerase epsilon (POLE) mRNA expression in clear cell renal cell carcinoma (ccRCC) compared with normal tissues. **(A)** POLE mRNA expression was significantly higher in patients with ccRCC compared with those of normal tissues (The Cancer Genome Atlas [TCGA] database). **(B–E)** Four test cohorts provided differential RNA-seq data in ccRCC and normal samples. The results suggested significantly elevated POLE expression levels in ccRCC tissues compared with normal tissues. The test cohorts were as follows: Gumz Renal (*n* = 10; *p* = 0.043), Cutcliffe Renal (*n* = 14; *p* = 0.011), Lenburg Renal (*n* = 9; *p* = 0.006), and Jones Renal (*n* = 23; *p* = 0.035).

To determine whether POLE expression is related to the occurrence and prognosis of ccRCC, we first examined five independent ccRCC datasets with available RNA sequencing data from TCGA and Oncomine databases. First, analysis of TCGA-kidney renal cell carcinoma (KIRC) dataset (*n* = 523) revealed that POLE mRNA expression in ccRCC tissues was significantly upregulated compared with that in normal tissues (Figure 1A). We then analyzed four test cohorts with enrolled differential RNA-seq data for ccRCC and normal samples. The analyses detected significantly elevated POLE expression levels in ccRCC compared with normal samples in the Gumz Renal (*n* = 10; *p* = 0.043), Cutcliffe Renal (*n* = 14; *p* = 0.011), Lenburg Renal (*n* = 9; *p* = 0.006), and Jones Renal (*n* = 23; *p* = 0.035) cohorts (Figures 1B–E). Overall, POLE expression is normally highly expressed in ccRCC tumor tissues compared with adjacent normal tissues.

### High Expression of Polymerase Epsilon Is Associated With Shorter Survival of Patients With Clear Cell Renal Cell Carcinoma

**FIGURE 2:**
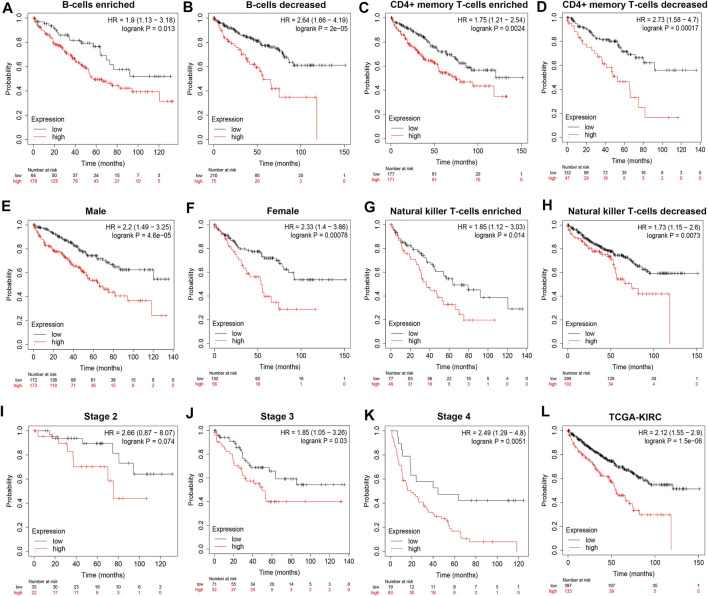
High expression of polymerase epsilon (POLE) is associated with poor survival of clear cell renal cell carcinoma (ccRCC) patients. **(A–L)** Subgroup survival analysis, including clinicopathological characteristics and abundance of immune cells in 533 patients with ccRCC (The Cancer Genome Atlas [TCGA] cohort), indicated that high POLE expression is significantly associated with poor prognosis.

To demonstrate independent prognostic implications of POLE expression, we performed subgroup survival analysis, including analyses of clinicopathological characteristics and abundance of immune cells in 533 patients with ccRCC in TCGA data. Patients with high POLE expression experienced significantly shorter OS than patients with lower expression (Figures 2A–K). Furthermore, high POLE expression was significantly associated with poor prognosis (Figure 2L). Therefore, POLE, which is highly expressed in ccRCC tissues, may be used as a promising biomarker for predicting prognosis.

### High Expression of Polymerase Epsilon in Pan-Cancers Compared With Adjacent Normal Tissues

**FIGURE 3:**
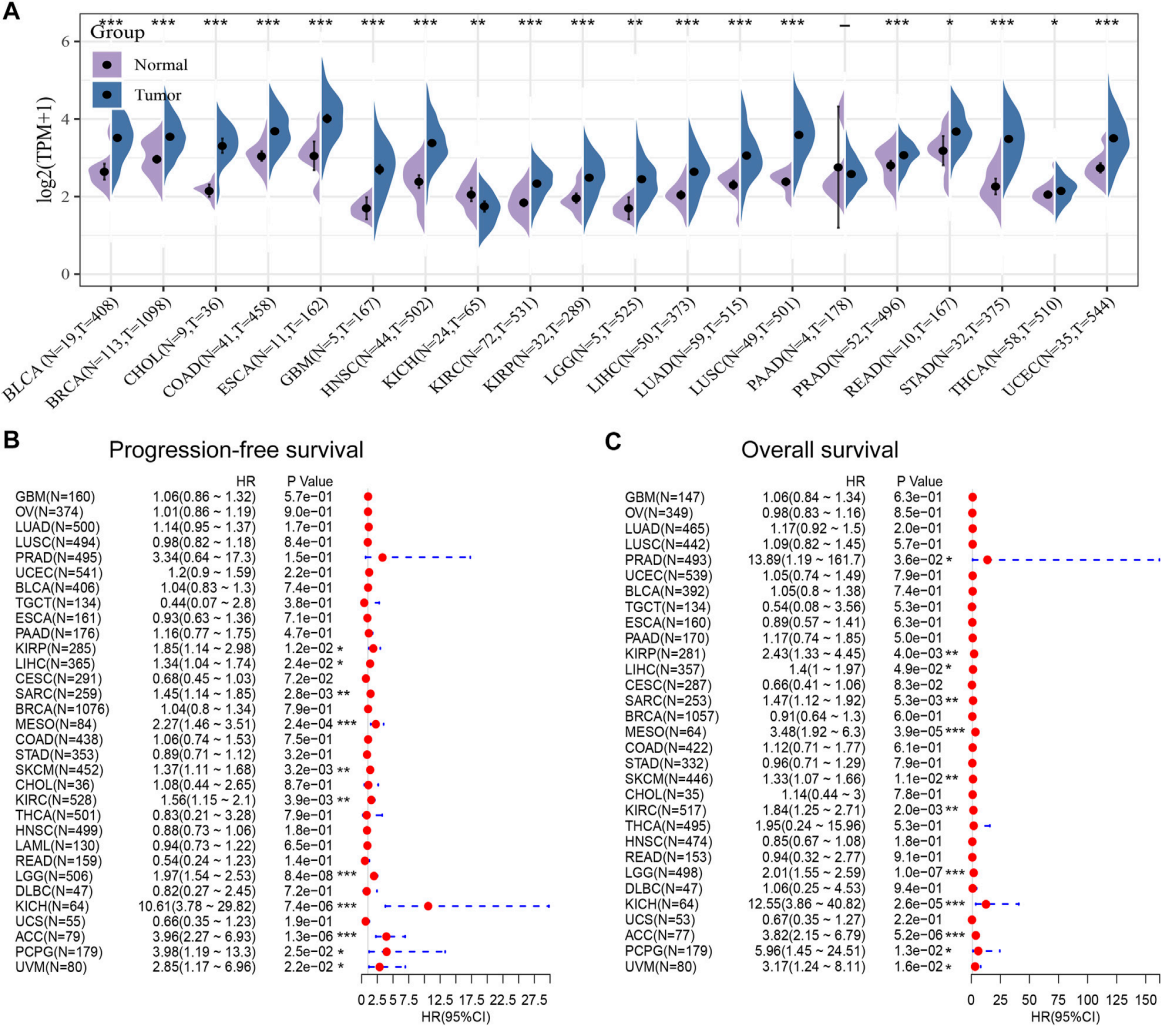
High expression of polymerase epsilon (POLE) in pan-cancers compared with adjacent normal tissues. **(A)** POLE expression levels between pan-cancer and normal tissues based on The Cancer Genome Atlas (TCGA) data. The differential expression of POLE between cancer tissue and adjacent normal tissue was common in pan-cancer, and the expression of POLE was mainly higher in tumor tissue. **(B, C)** Cox regression analysis suggests that POLE expression is closely linked with overall survival (OS) in many cancers.

Next, we compared POLE expression levels between pan-cancer and normal tissues based on TCGA data. The differential expression of POLE between cancer tissue and adjacent normal tissue was common in pan-cancer, and the expression of POLE was high mainly in tumor tissue (Figure 3A). Therefore, to explore the prognostic implications of POLE mRNA expression in cancers, we performed Cox regression analysis to identify the effect of POLE expression on the progression-free survival and OS of pan-cancer (Figures 3B,C). POLE expression was closely linked with OS in many cancers such as Adrenocortical carcinoma (ACC) (hazard ratio (HR) = 3.96; *p* = 1.3e−06), Brain Lower Grade Glioma (LGG) (HR = 1.97; *p* = 8.4e−08), and Skin Cutaneous Melanoma (SKCM) (HR = 1.37; *p* = 0.0032); and DSS in ACC (HR = 3.82; *p* = 5.2e−06), LGG (HR = 2.01; *p* = 1.0e−07), Mesothelioma (MESO) (HR = 3.48; *p* = 3.9e−05), and Kidney Chromophobe (KICH) (HR = 12.55; *p* = 2.6e−05).

### DNA Variant Landscape of Polymerase Epsilon Mutation Profiles in Clear Cell Renal Cell Carcinoma

**FIGURE 4:**
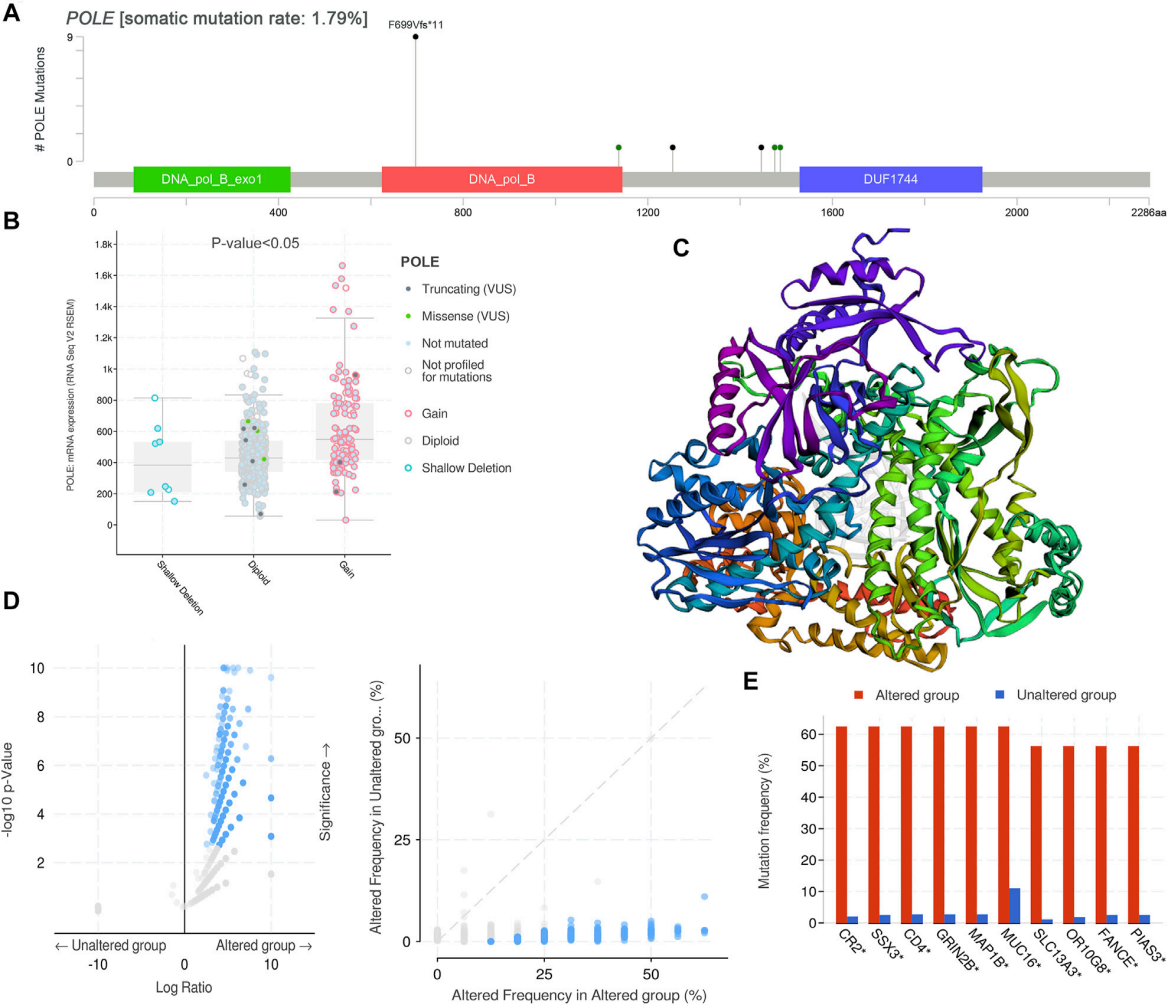
DNA variation landscape of polymerase epsilon (POLE) mutation profiles in clear cell renal cell carcinoma (ccRCC). **(A)** The mutations mainly occur in exon DNA_pol_B and some introns. **(B)** The expression of POLE was significantly increased in the gain group, the expression of POLE in diploid cells was lower, and mRNA expression levels were significantly decreased in the POLE shallow-deletion gene group. **(C, D)** Screening for significant difference in gene expression between POLE-altered and POLE-unaltered genes. **(E)** The top 10 upregulated genes ranked by mutation frequency are CR2, SSX3, GRIN2B, MAP1B, MUC16, SLC13A3, OR10G8, FANCE, and PIAS3.

After discovering the important role of POLE expression associated with poor prognosis, we investigated the potential effects of POLE mutations. The mutations mainly occur in exon DNA_pol_B and certain introns (Figure 4A). The expression of POLE was significantly increased in the copy number gain group. In contrast, the expression of the POLE in diploid cells was lower, and the mRNA expression level was significantly decreased in the POLE-shallow deletion gene group (*p* < 0.05) (Figure 4B). Therefore, mutation of POLE significantly correlated with mRNA expression levels as well as the progression and prognosis of ccRCC. Nevertheless, the OS rates of the POLE-altered and POLE-unaltered groups were not significantly different, suggesting that the POLE mutation will not serve as a significant predictive marker for OS.

**FIGURE 5:**
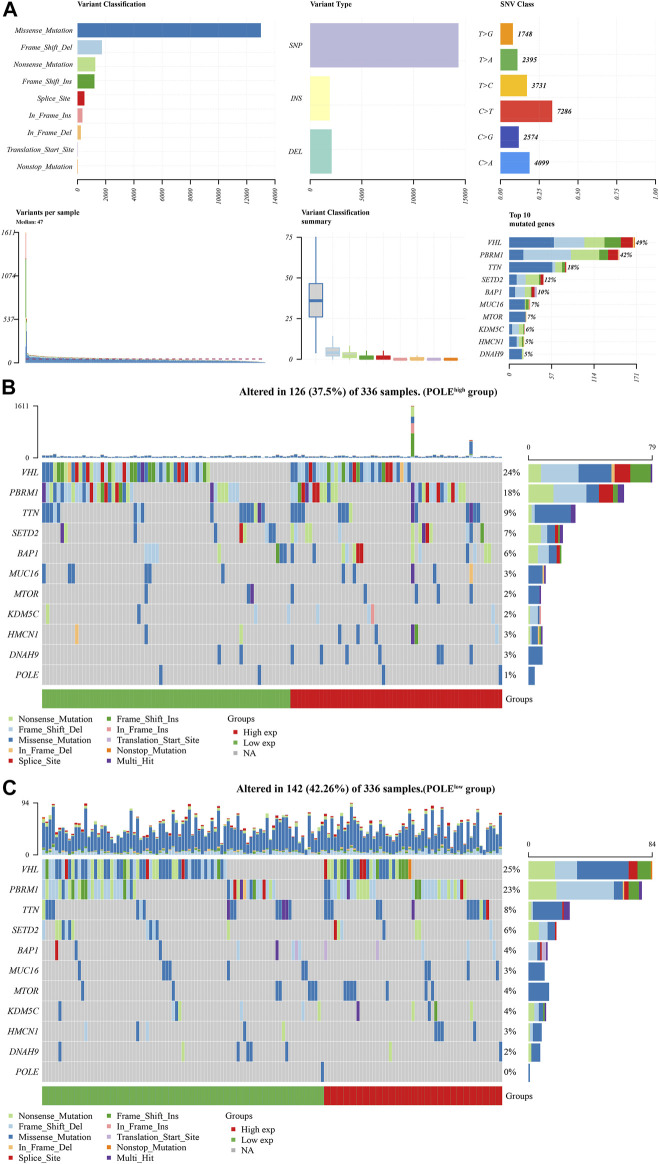
Differential mutation landscape between different polymerase epsilon (POLE) expression profiles in clear cell renal cell carcinoma (ccRCC). **(A)** The variant classifications, variant types, and single-nucleotide variant (SNV) classes of POLE are summarized. SNVs of the top 11 mutated POLE genes. **(B, C)** The somatic landscapes of cells with high and low POLE expression, with the top 10 genes ordered according to mutation frequency. In particular, the PBRM1 mutation rate was 23% in the POLE^low^ group vs. 18% in the POLE^high^ group.

There was a significant difference in gene expression levels between POLE-altered and POLE-unaltered genes (Figures 4C,D). The top 10 upregulated genes ranked according to mutation frequency were as follows: CR2, SSX3, GRIN2B, MAP1B, MUC16, SLC13A3, OR10G8, FANCE, and PIAS3 (Figure 4E). The variant classifications, types, and single-nucleotide variant (SNV) classes of POLE genes are summarized in Figure 5A. The SNVs harbored by the top 11 mutated POLE genes are shown as well. The somatic gene landscapes associated with high and low POLE expression are presented in Figures 5B,C, with the top 10 genes ordered according to mutation frequency. In particular, the PBRM1 mutation rates of the POLE^low^ and POLE^high^ groups were 23% and 18%, respectively.

### Validation of Differential Polymerase Epsilon Expression in the Fudan University Shanghai Cancer Center Cohort and Its Prognostic Value

**FIGURE 6:**
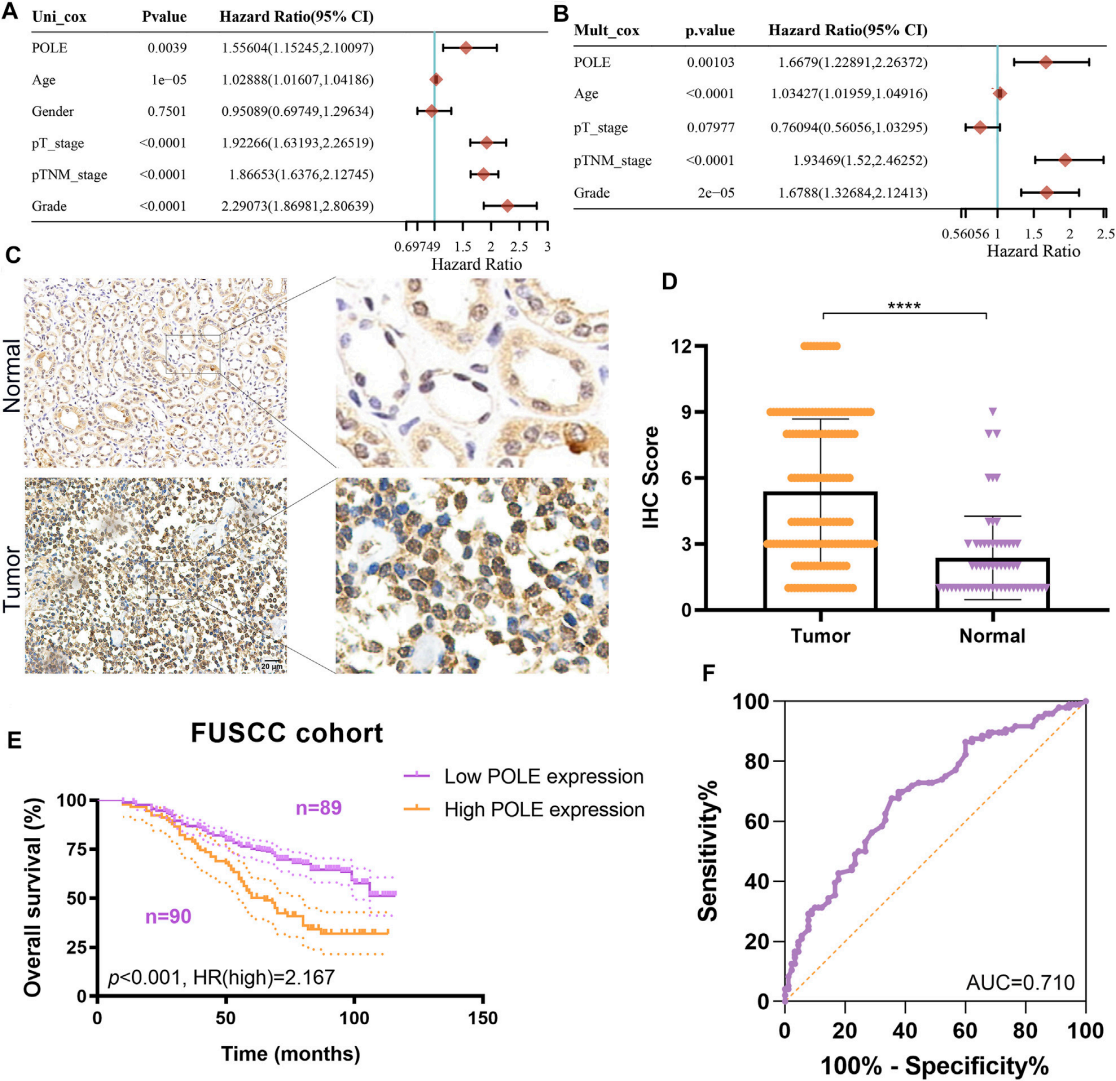
Validation of differential polymerase epsilon (POLE) expression and its prognostic value in the Fudan University Shanghai Cancer Center (FUSCC) cohort. **(A,B)** Univariate and multivariate Cox regression analyses was performed to reveal independent prognostic value of POLE expression for the outcomes of patients with ccRCC. **(C)** IHC analysis of samples from the FUSCC cohort showing that the expression of POLE was significantly higher in ccRCC than in normal kidney tissues. **(D)** The IHC scores of POLE expression in ccRCC tissues and normal tissues . **(E)** Prognostic implications of POLE expression in patients with 179 ccRCC in the FUSCC cohort. **(F)** ROC curve indicating the sensitivity and specificity of the independently diagnostic and prognostic value of POLE expression.

To optimize predictive models and enhance clinical translational efficiency, we used a nomogram to validate the prognostic implications of POLE expression. Univariate and multivariate Cox regression analyses revealed that POLE was the most clinically significant marker for predicting the outcomes of patients with ccRCC (*p* < 0.01) (Figures 6A,B) To verify whether the expression of POLE was higher in ccRCC samples compared with normal kidney tissue, we performed IHC analysis and found that expression of POLE was higher in ccRCC than in normal kidney tissues (Figure 6C). The IHC score proved that POLE expression was significantly elevated in ccRCC compared with normal tissues (*p* < 0.0001) (Figure 6D).

We subsequently explored the prognostic implications of POLE expression in 179 patients with ccRCC of the FUSCC cohort. Higher expression of POLE was significantly associated with worse OS (*p* < 0.001, HR = 2.167) (Figure 6E). The receiver operating characteristic (ROC) curve indicated high sensitivity and specificity of the independently diagnostic and prognostic values of POLE expression (area under the ROC curve (AUC) = 0.710) (Figure 6F). Taken together, we revealed that POLE, which is highly expressed in ccRCC tissues, is a promising prognostic biomarker, and it is worthy of further exploration at the transcriptome and proteome levels.

### Construction of a Protein–Protein Interaction Network and Functional Enrichment Analysis

**FIGURE 7:**
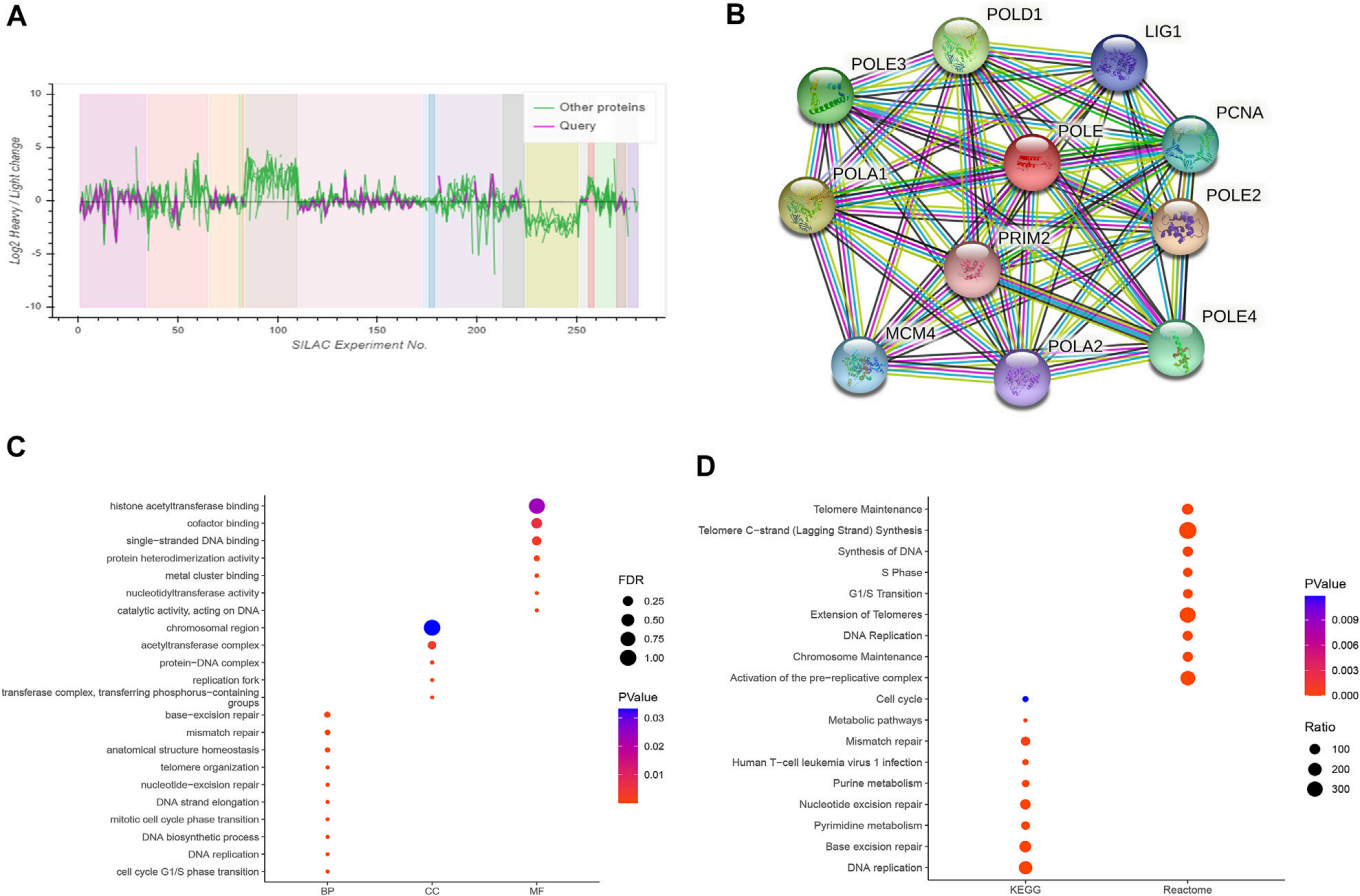
Construction of a protein–protein interaction (PPI) network and functional enrichment analysis. **(A)** Analysis of 11 proteins coordinately regulated with polymerase epsilon (POLE). **(B)** The PPI comprised 11 hub proteins, as follows: POLE, POLE2, POLE3, POLE4, PCNA, LIG1, POLD1, POLA1, POLA2, MCM4, and PRIM2. **(C, D)** Gene Ontology (GO) functional annotation, including biological process, cellular components, and molecular function, and enrichment analyses of Kyoto Encyclopedia of Genes and Genomes (KEGG) and Reactome pathways were used to characterize the hub proteins. We found critical factors such as DNA replication and DNA biosynthetic process in biological process (BP), replication fork and protein–DNA complex in cellular component (CC), nucleotidyltransferase activity and metal cluster binding in molecular function (MF), base excision repair and nucleotide excision repair in KEGG, and extension of telomeres in Reactome.

Based on the cutoff threshold, we obtained 11 significantly coregulated proteins (Figure 7A). Then, we constructed PPI network and found the 11 critical gene panels, consisting of POLE, POLE2, POLE3, POLE4, PCNA, LIG1, POLD1, POLA1, POLA2, MCM4, and PRIM2 (Figure 7B). To analyze the function of these genes, we conducted pathways enrichment including GO: BP, CC, MF, and KEGG (Figures 7C,D). We found critical factors like DNA replication and DNA biosynthetic process in BP, replication fork and protein–DNA complex in CC, nucleotidyltransferase activity and metal cluster binding in MF, base excision repair and nucleotide excision repair in KEGG, and of Telomeres in Reactome.

### Assessment of the Correlation Between Polymerase Epsilon Expression and Immune Cells

**FIGURE 8:**
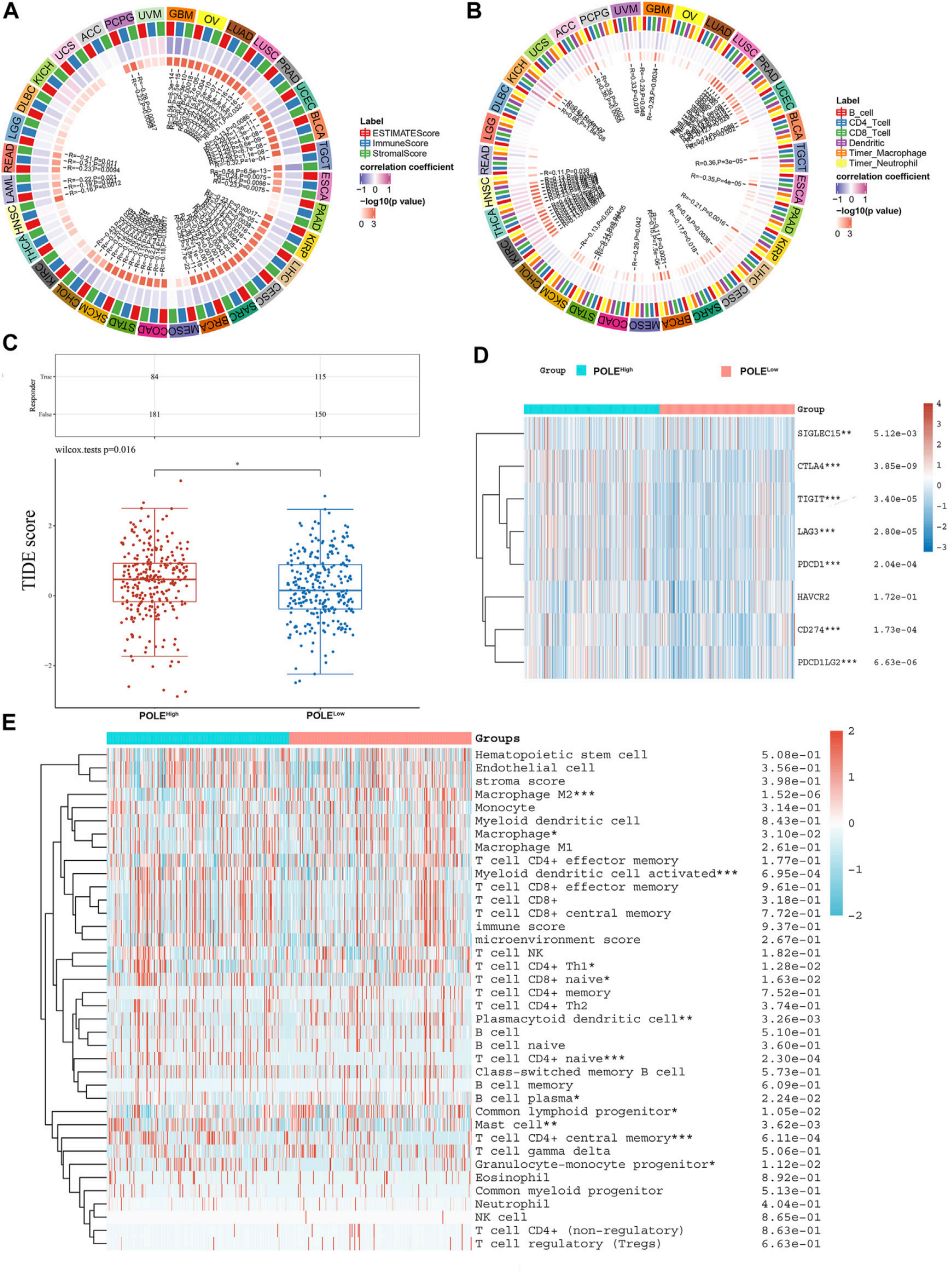
Correlation between polymerase epsilon (POLE) expression and immune cells. **(A)** Significant association was found between POLE expression with estimate score, immune score, and stromal score of pan-cancer. **(B)** Immune cells, including B cells, T cells, dendritic cells, macrophage, and neutrophil cells, were markedly associated with the POLE expression of cancers in varying degrees, especially in clear cell renal cell carcinoma (ccRCC). **(C)** The TIDE algorithm is used to evaluate two different tumor immune escape mechanisms and was developed in POLE^high^ compared with low POLE expression group in ccRCC cohort. **(D)** Analyses of the comprehensive correlation between immune cells and pan-cancer. ccRCC mainly involved CD56bright natural killer cells and activated CD4 T cells. There was a significant relationship between immune cells and ccRCC, indicated by the expression of immune checkpoint molecules such as PDCD1, CTLA4, CD86, and TNFSF9. **(E)** The heat map shows that high expression of POLE was closely associated with M2 macrophages, activated myeloid dendritic cells, plasmacytoid dendritic cells, Th1 CD4^+^ T cells, naive CD4^+^ T cells, mast cells, and granulocyte-monocyte progenitors.

We show here that high expression of POLE significantly correlated with worse prognosis of patients with ccRCC and that high expression of POLE promoted the function of the immune system. There was a significant association among POLE expression with the ESTIMATE score, immune score, and stromal score of pan-cancer (Figure 8A). Moreover, many immune cell subsets, including B cells, T cells, dendritic cells, macrophages, and neutrophils, were significantly associated with POLE expression in cancers to varying degrees, particularly in ccRCC (Figure 8B). The TIDE algorithm is used to evaluate two different tumor immune escape mechanisms. Here, we applied TIDE to compare POLE^high^ and POLE^low^ expression groups in the ccRCC cohort (Figure 8C). We found that the TIDE score was significantly higher in the POLE^high^ group compared with the POLE^low^ group, suggesting that patients with high POLE expression are resistant to treatment with ICTs.

Furthermore, significant relationships between immune cells and ccRCC involved immune checkpoint molecules such as PDCD1, CTLA4, CD86, and TNFSF9 (Figure 8D). The heat map (Figure 8D) shows that high expression of POLE was closely related to M2 macrophages, activated myeloid dendritic cells, plasmacytoid dendritic cells, Th1 CD4^+^ T cells, naive CD4^+^ T cells, mast cells, and granulocyte-monocyte progenitors (Figure 8E). Moreover, in >12,000 pan-cancer samples, we found that POLE expression significantly predicted an immune-suppressive microenvironment because of the markedly negative correlation with abundance of many types of infiltrating immune cells (Supplementary Figure S1). Overall, POLE expression significantly correlated with the infiltration of tumors with immune cells and provided an immune-suppressive tumor microenvironment.

### Potential Functions of Polymerase Epsilon in Progression and Immune Escape of Clear Cell Renal Cell Carcinoma

We screened DEGs related to POLE and performed functional annotations to identify the potential role of POLE in patients with ccRCC. First, we screened potentially functionally related genes according to the high and low of POLE expression (Supplementary Figure S2A). We subsequently used hierarchical cluster analysis to identify the DEGs associated with POLE in ccRCC and analyzed in detail their differences between tumor and normal tissue (Supplementary Figure S2B). Next, we conducted selective enrichment of the KEGG signaling pathways and GO terms (including BPs, CCs, and MFs) and analyzed the selected DEGs to demonstrate their main biological effects on POLE (Supplementary Figures S2C–2D). These analyses revealed that DEGs were mainly related to sister chromatid segregation, regulation of small GTPases, regulation of PH (potential of hydrogen potential of hydrogen), organic acid transport, and monovalent inorganic cation homeostasis. In the KEGG signaling pathway, DEGs were mainly related to *Yersinia* infection, Th17 cell differentiation, and the TNF signaling pathway.

Next, we investigated the biological functional annotations of POLE associated with regulating tumor progression and assessed the correlations between POLE expression and significant markers in the processes mediated by the hallmarks mentioned above. These analyses predicted that POLE expression promotes tumor progression and immune escape of ccRCC via regulation of combined inhibition by the Notch and JAK/STAT pathways as well as by PD-L1 expression (Supplementary Figure S3).

## Discussion

Here, we performed clinicopathological, IHC, and survival analyses to investigate the potential prognostic value of POLE in ccRCC at the mRNA and protein levels. Elevated POLE expression significantly correlated with worse prognosis of ccRCC. Therefore, the expression of POLE may serve as a valuable biomarker for the diagnosis and prognosis of ccRCC. Furthermore, high POLE expression levels were significantly associated with higher grade, advanced stage, worse outcomes, and high risk of recurrence.

To evaluate the possible functions of POLE of ccRCC, we constructed a PPI network and performed functional enrichment analysis. The results suggest the nature of pathways mediated by POLE in association with other signaling proteins, illustrating the relationship between high POLE expression and ccRCC through changes in DDR. Therefore, this evidence led us to further explore the role of ICT in the regulation of POLE expression to restore the DDR function of patients with ccRCC. Importantly, POLE was involved in the infiltration of the tumor microenvironment of pan-cancer, which sheds novel insights into drug development and may improve the efficiency of clinical therapies.

POLE influences oncogenesis and disease progression by regulating tumor promoter genes and DDR, thus guiding targeted anticancer therapy (Bellido et al., 2016). POLE mutations help promote mutagenesis (Park and Pursell, 2019). DDR pathways comprise multiple interconnected cellular signaling pathways, which coordinate a cascade of events to respond to DNA damage (Tian et al., 2015; Brandsma et al., 2017). As a functional binding complex required for the DNA double-strand break repair function of the DDR pathway, Rad50-MRE11-NBN was identified as a tumor promoter and drug-sensitive prodding agent in many cancers. (Wang et al., 2019a; Xu et al., 2020b). Furthermore, many genomic biomarkers associated with homologous recombination deficiency confer benefits upon patients administered PARP inhibitors and long-term clinical practice (long-term PARP inhibitor therapy, other therapeutics, palliative care, etc.) (Pai Bellare et al., 2021; Setton et al., 2021; Verma et al., 2021).

Previous studies investigating the mechanism of the transcriptional regulation of POLE expression and mutations in carcinogenesis show that POLE or POLD1 mutations serve as negative prognostic markers and may predict a survival benefit from ICI therapy across diverse cancers (Wang et al., 2019b). Moreover, POLE mutations were identified as a significant signature predicting OS and response to immunotherapy of Chinese patients with endometrial carcinomas (He et al., 2020). Furthermore, the tumor phenotypes and POLE/POLD1 expression levels correlate with tumor mutation burden and tumor driver gene expression according to an analysis of a panel of mutations in 1,392 Chinese patients with cancer (Yao et al., 2019). These studies provide theoretical and practical justifications for evaluating the status of POLE/POLD1 of patients administered immunotherapy.

In the present study, patients with ccRCC with POLE mutations did not experience significant differences in survival benefits as compared with those without POLE mutations, likely because of the low mutation frequency of ccRCC. Therefore, the long-term prognostic effect of POLE mutations on patients with ccRCC should be explored in larger-scale, worldwide cohorts. For example, evidence shows that POLE activates antitumor immunity and improves the tumor microenvironment, consistent with our present findings. Although the effects of POLE mutations on the OS of patients with ccRCC were not significant, patients with altered POLE expression harbor genes with higher mutation frequencies. Thus, the disadvantageous and beneficial relationships between POLE mutations and prognosis of ccRCC require further study. Moreover, Cox regression analysis elucidated a correlation between POLE expression and OS in pan-cancer, proving the universality of the influence of POLE expression on cancers.

A limitation of the present study is that IHC analysis was not accompanied by functional analyses, studies of an appropriate animal model, or both. Thus, direct evidence must be acquired to discern the effect of POLE on the malignant behaviors of ccRCC. Furthermore, future studies must identify the underlying mechanism of POLE function in ccRCC.

The most significant findings of the present study are as follows: first, we analyzed independent ccRCC cohorts and a real-world FUSCC cohort. Second, we demonstrated a powerful role of POLE expression associated with the prognosis of ccRCC. Third, we validated the assumption that mRNA and protein levels accurately confirm the association between POLE expression and prognosis of ccRCC. Moreover, >12,000 tumor samples from TCGA data were analyzed, extending the findings of studies on ccRCC to pan-cancer. Fourth, functional enrichment analysis and the role of POLE in the immune-cell infiltration of the tumor microenvironment were closely linked to cancer treatment and the development of targeted drugs.

## Conclusion

The present study provides compelling data supporting the conclusion that elevated POLE expression is significantly linked with poor outcomes and the immune-suppressive tumor microenvironment of ccRCC. Together, these findings suggest that POLE will serve as a biomarker, guiding molecular diagnosis and identifying novel individual therapeutic strategies for patients with advanced ccRCC.

## Data Availability

The raw data supporting the conclusion of this article will be made available by the authors, without undue reservation.
