# Supplementary figures and images for: Protumorigenic Role of Elevated Levels of DNA Polymerase Epsilon Predicts an Immune-Suppressive Microenvironment in Clear Cell Renal Cell Carcinoma
Source: Front Genet. 2021 Dec 7;12:751977. doi: 10.3389/fgene.2021.751977 (PMC8689073; doi:10.3389/fgene.2021.751977)

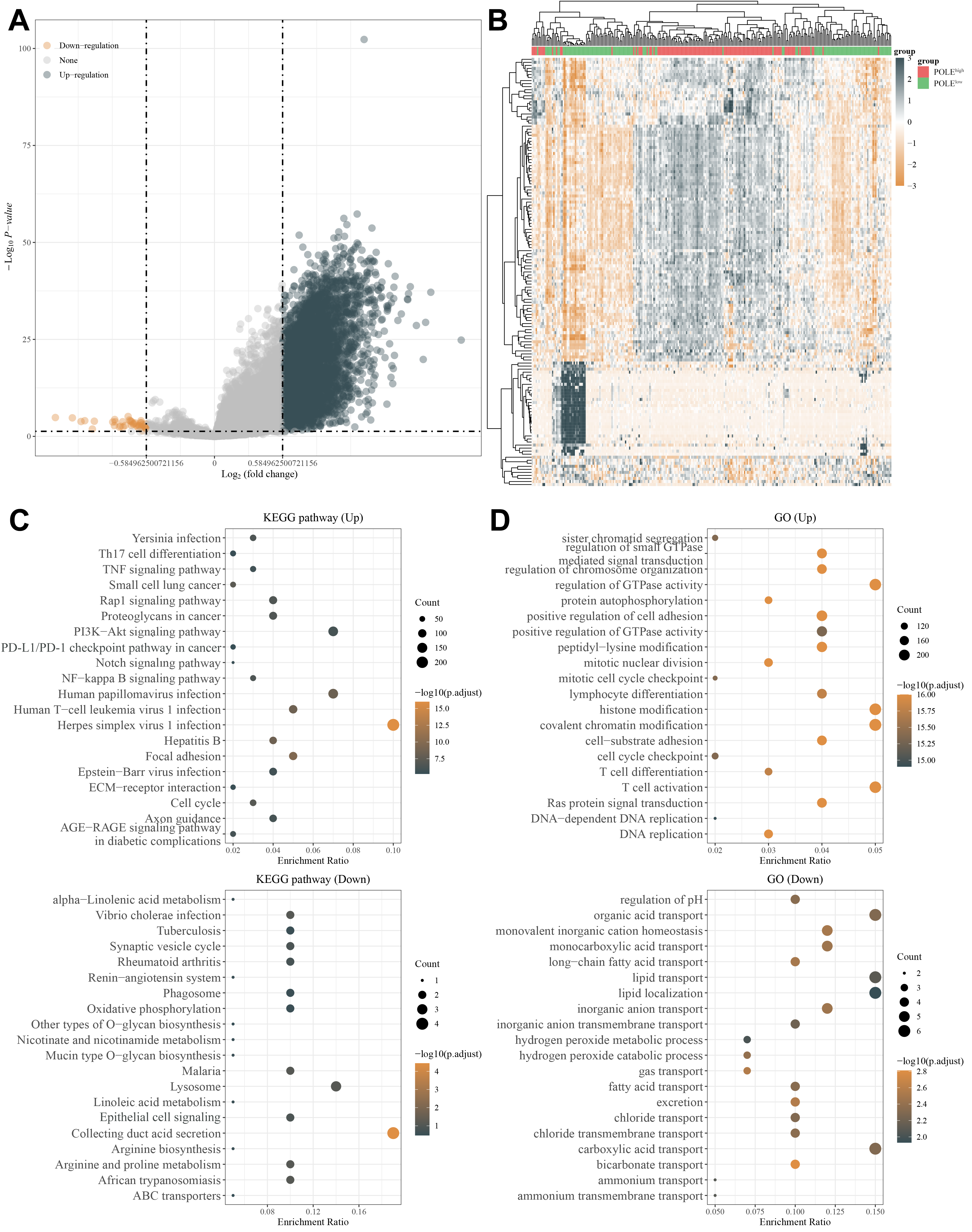

Supplement: Supplementary file 1 [file Image2.TIF]

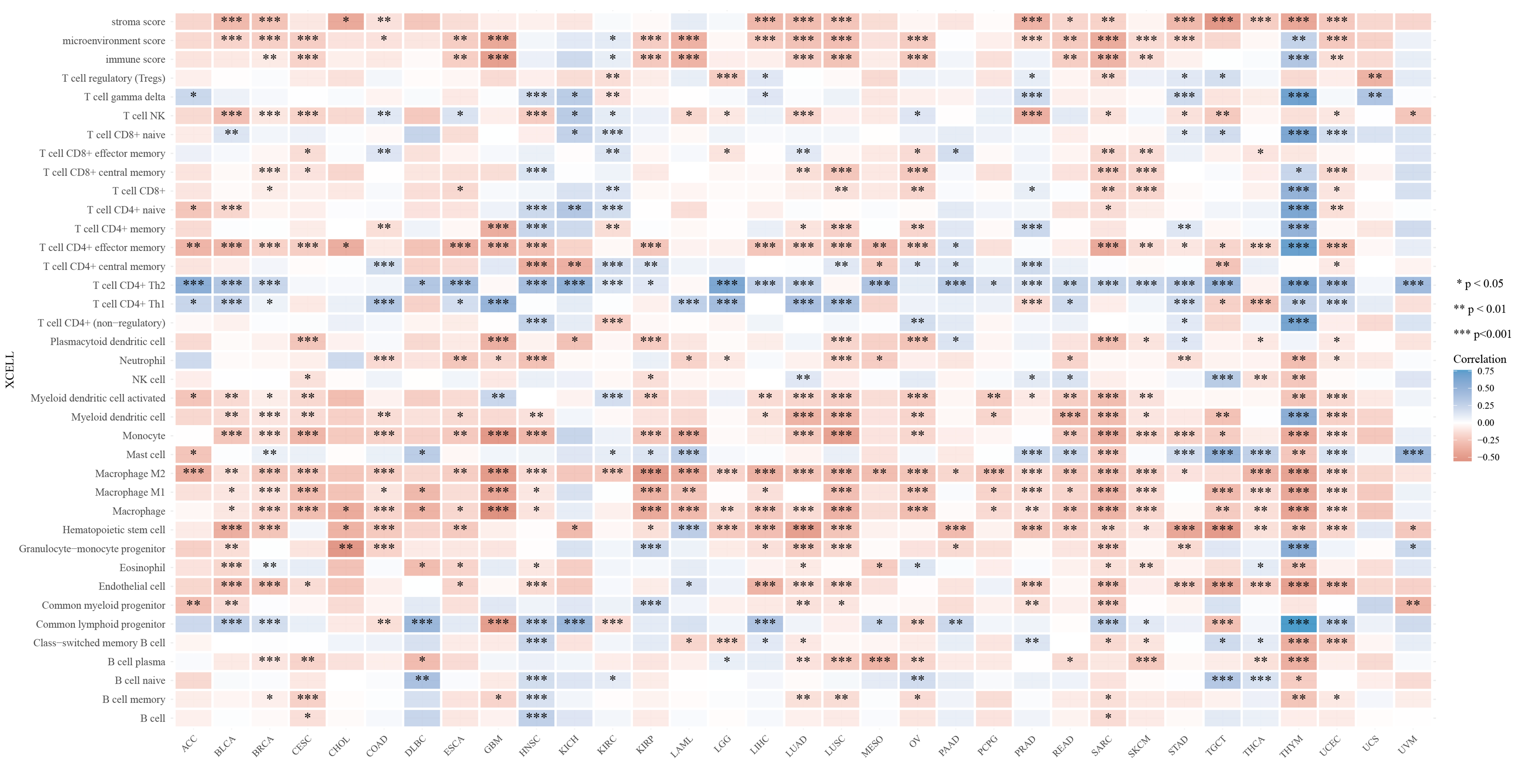

Supplement: Supplementary file 2 [file Image1.TIF]

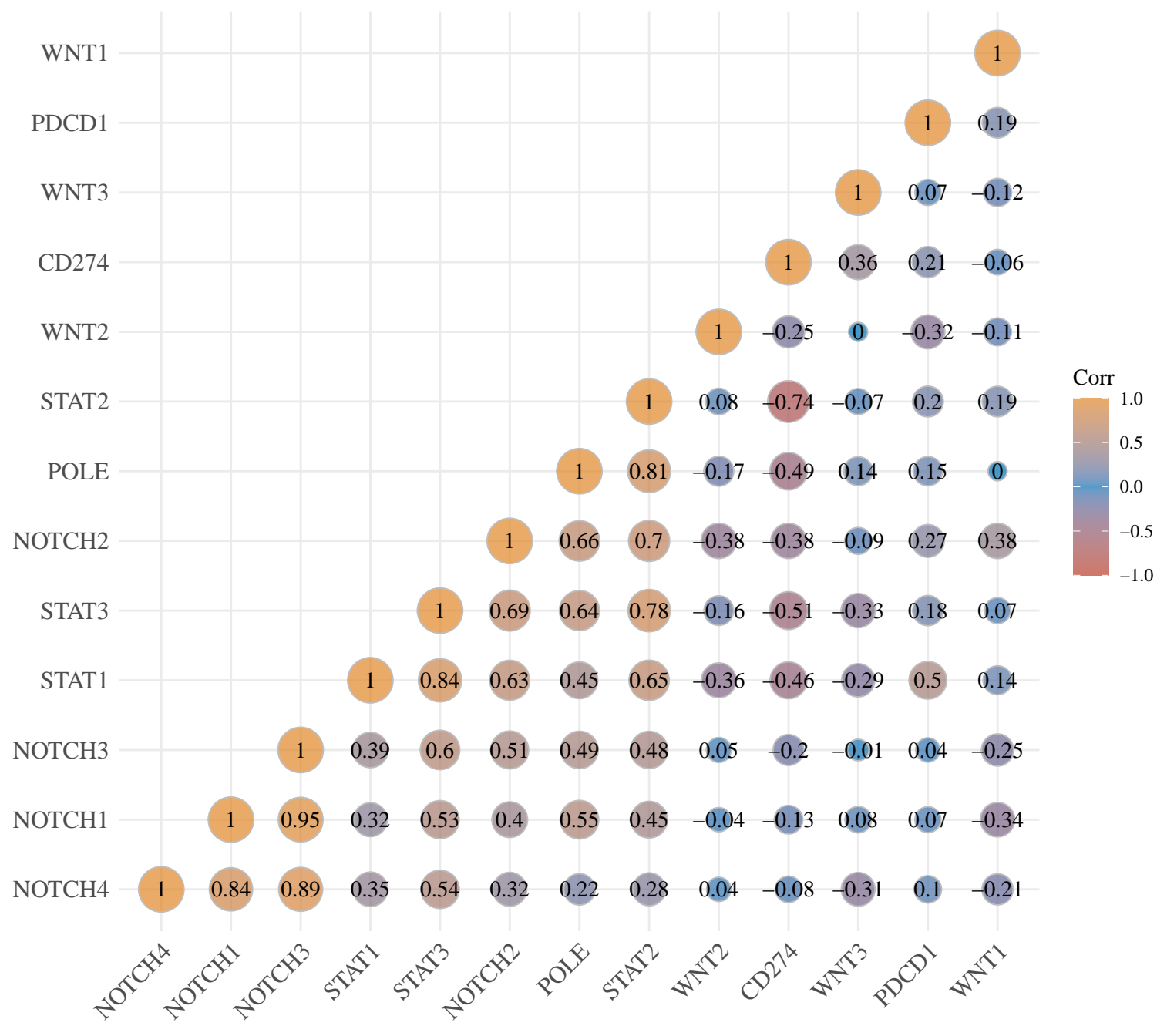

Supplement: Supplementary file 3 [file DataSheet1.PDF]
